# A Systematic Review and Meta-Analysis of Risk Factors for Sexual Transmission of HIV in India

**DOI:** 10.1371/journal.pone.0044094

**Published:** 2012-08-28

**Authors:** Paul Arora, Nico J. D. Nagelkerke, Prabhat Jha

**Affiliations:** 1 Division of Epidemiology, Dalla Lana School of Public Health, University of Toronto, Toronto, Ontario, Canada; 2 Department of Community Medicine, United Arab Emirates University, Al-Ain, Abu Dhabi, United Arab Emirates; 3 Centre for Global Health Research, Li Ka Shing Knowledge Institute, St. Michael's Hospital, Toronto, Ontario, Canada; University of Washington, United States of America

## Abstract

**Background:**

Approximately 2.4 million people are living with HIV in India. This large disease burden, and potential for epidemic spread in some areas, demands a full understanding of transmission in that country. We wished to quantify the effects of key sexual risk factors for HIV infection for each gender and among high- and low-HIV risk populations in India.

**Methodology:**

We conducted a systematic review of sexual risk factors for HIV infection from 35 published studies. Risk factors analyzed were: male circumcision/religion, Herpes Simplex Virus 2, syphilis, gonorrhoea, genital ulcer, multiple sexual partners and commercial sex. Studies were included if they met predetermined criteria. Data were extracted and checked by two researchers and random-effects meta analysis of effects was conducted. Heterogeneity in effect estimates was examined by I^2^ statistic. Publication bias was tested by Begg's test and funnel plots. Meta regression was used to assess effect modification by various study attributes.

**Results:**

All risk factors were significantly associated with HIV status. The factor most strongly associated with HIV for both sexes was HSV-2 infection (OR_men_: 5.87; 95%CI: 2.46–14.03; OR_women_: 6.44; 95%CI: 3.22–12.86). The effect of multiple sexual partners was similar among men (OR = 2.46; 95%CI: 1.91–3.17,) and women (OR = 2.02; 95%CI: 1.43–2.87) and when further stratified by HIV-risk group. The association between HSV-2 and HIV prevalence was consistently stronger than other STIs or self-reported genital ulcer. If the strong associations between HSV-2 and HIV were interpreted causally, these results implied that approximately half of the HIV infections observed in our study population were attributable to HSV-2 infection.

**Conclusions:**

The risk factors examined in our analysis should remain targets of HIV prevention programs. Our results confirm that sexual risk factors for HIV infection continue to be an important part of Indian HIV epidemic 26 years after it began.

## Introduction

Approximately 2.4 million people live with HIV/AIDS in India today [1]. While this represents a relatively small proportion of India's 1.2 billion people, a large absolute burden and the potential for epidemic spread demands a full understanding of HIV transmission in that country. It is important to study risk factors because the nature of the epidemic should be taken into consideration when choosing prevention approaches. Thailand experienced a very successful HIV prevention campaign in the 1990s by achieving a high rate of condom use for high-risk sex contacts in brothels[2]. HIV incidence subsequently fell but due to a combination of the drying up of the free condom supply, due to the Asiatic Crisis of 1997, and the shift of sex work typology from brothel to home- and street-based, HIV incidence began to increase again.

Previous studies on HIV in India have identified heterosexual sex (chiefly through commercial sex between male clients and female sex workers) as the primary driver of HIV incidence in the population [3], [4], [5], [6], [7], [8]. Key risk factors that have been identified in the literature are use of, or engaging in, commercial sex work [8], [9], bacterial and viral sexually transmitted infections (STI) [5], [7], [10], numbers of sex partners [11], [12], [13], [14] and male circumcision [6], [11], [15], [16]. These are examples of “proximal” risk factors [17]. Distal risk factors have been identified including: proximity to brothel, highways and impaired access to STI clinical services[17], [18]. In this study we focus on proximal factors as important targets for interventions.

The Indian HIV epidemic exhibits a large amount of geographic heterogeneity in terms of HIV risk [19], [20]. We were interested in understanding the effects of these risk factors, and estimating their size, for each gender and in different epidemiologic settings (populations at higher or lower risk of HIV infection due to their behaviours and different types of STIs such as bacterial versus viral), to improve understanding of transmission dynamics [21], [22]. Documenting and explaining this variation would improve our understanding of the epidemic in India. We know of no published systematic review that has quantified the effects of these risk factors in India.

## Methods

### Ethics Statement

N/A

### Searching

Published studies were identified by searching electronic databases. The protocol for this review is unregistered. We searched three databases: Web of Science, EMBASE and Medline on June 9^th^ 2011. We searched (and exploded) the terms: “HIV”, “delta retrovirus”, “Disease Transmission” (including infectious/horizontal transmission), “Risk Factors”, “sexually transmitted infection” or disease, “herpes” or HSV and “India”. We combined these terms by merging results for HIV/delta retrovirus, any combination of the subsequent search terms, save India, and the last term. Results were limited to English language studies (due to logistic constraints) and those published from 1986 onwards (the year that HIV was first identified in India).

### Validity assessment

Quality review was done using an approach recommended by the MOOSE group guidelines for meta-analysis of observational studies [23] and Greenland *et. al.* [*24*]. Briefly, all eligible studies were included (if they met minimal inclusion criteria described below) and the influence of key factors, selected *a priori*, were assessed using meta-regression. Where possible stratification of study results by these factors was done (gender, HIV-risk population, study year, research design and state). Seven specific characteristics were used to determine sufficient quality for inclusion. These factors included: biological confirmation of STIs, biological confirmation of HIV status following WHO guidelines, clear description of HIV-risk population, description of study population selection methods, provision of raw numbers of subjects exposed and unexposed by HIV status, separate reporting of unadjusted and adjusted estimates of effect and reporting a measure of variance around the effect estimate (variance, standard error or confidence intervals).

### Data abstraction

Data was abstracted and entered twice (two people) into a standard Excel template and cross-checked by each data extractor. Where required data were likely available but not presented in a published included study, the study authors were contacted for the relevant data.

### Cases and Controls

Case (HIV-positive) status was determined by referring to WHO guidelines for screening in developing countries (two reactive enzyme-linked immunosorbent assays) (24). We included both prevalent and incident HIV cases. In the final complement of 35 studies, only four reported on incident HIV cases.

### Definition of exposures

We were interested in four broad sexual risk factors for HIV. These were STIs, male circumcision, genital ulcers and sexual behaviour. Sexual behaviour was sub-categorized into: paid sex (for men this was ever paying for sex while for females this was ever having been paid for sex) and lifetime numbers of sex partners (> = 2 *versus* 0–1 partners). Where lifetime sexual partnerships were not available, current/recent number of sexual partners was used instead. In India, male circumcision is almost exclusively practiced by Muslims[6]. Male circumcision status was collected as circumcision status or Muslim religion. Female Muslim religion was also collected. STIs were separated into biologically confirmed STIs and genital ulcer. STIs included in our analysis were: herpes virus 2 (HSV-2), syphilis (infection with *Treponema pallidum*) and gonorrhoea (*Neiserria gonorrhoea* infection). Information on stage of syphilis infection was not available for most studies. Genital ulcer was recorded as self-reported history of genital ulcer or history (ever) of diagnosis.

### Stratification by time, gender and risk population

Study populations were stratified by gender and HIV risk group. HIV risk populations were broadly categorized as “high” and “low” as per broad categories used by the National AIDS Control Organization in India [3]. High risk groups were: female sex workers (FSW), men who have sex with men (MSM), injecting drug users (IDU), clients of FSWs, STI clinic attendees, truckers and paid blood donors. Low risk groups were: antenatal clinic attendees, general population, non-STI hospital patients and voluntary blood donors.

### Statistical methods

Odds ratios (OR) were calculated for each risk factor and meta-analysis was conducted. To account for heterogeneity between study effect estimates, summary ORs were calculated using the random effects methods of DerSimonian and Laird. This method was chosen because we wished to make inferences about the effect of exposures beyond the population of studies observed in our analysis and account for between study variations in effect estimates [25]. Population attributable fractions were calculated for summary effect estimates. Heterogeneity in effects was tested using the I^2^ statistic, which measures the percentage of variation across studies due to heterogeneity rather than chance [26]. Publication bias was tested using Egger's test and visually assessed with funnel plots [27]. The influence of individual studies on summary effect measures for each risk factor was examined with influence plots (plots of summary effects with each study removed). Meta regression for the effects of gender, research design, study year, HIV risk population and methods of exposure and outcome measurement were done individually to assess effect modification. All analyses were done using Stata 12 (Houston, TX. USA).

## Results

**Figure 1:**
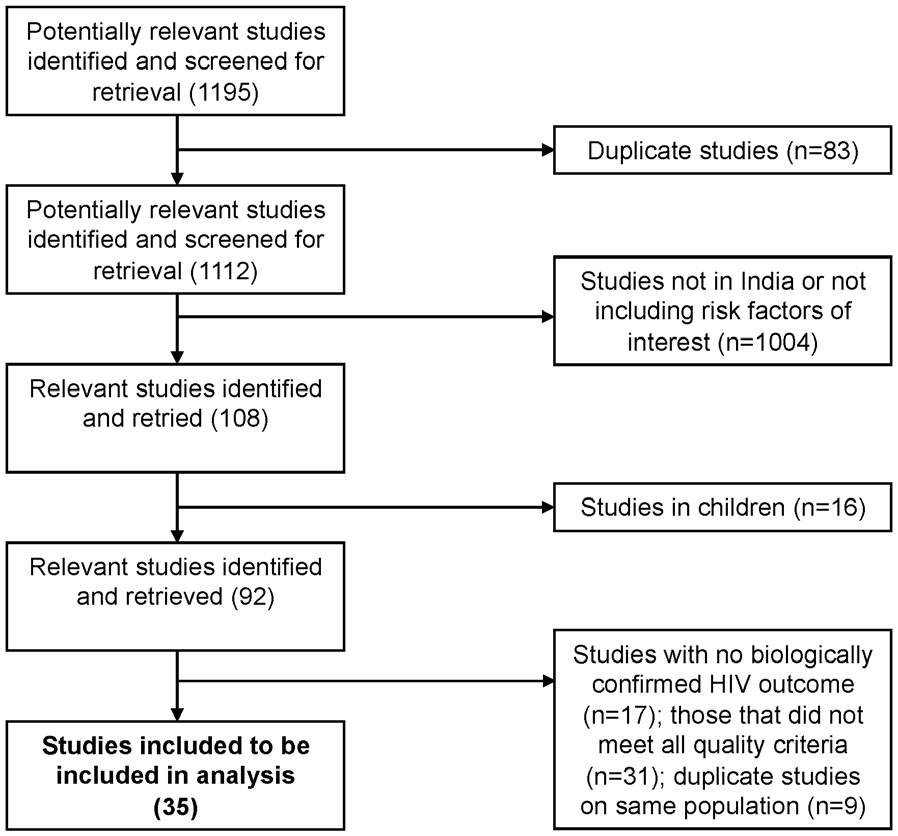
Flow of search strategy and included studies.

Our search strategy initially yielded 1195 results. The final dataset contained 35 studies. The final number of studies was arrived at as follows (diagrammed in **Figure 1**): After the removal of 83 duplicates (due to the use of multiple databases), 16 articles that dealt exclusively with children, and 1004 studies not conducted on Indian populations or not involving any of our pre-selected risk factors for HIV, 92 articles remained. After reviewing the methods section of these studies, 17 were removed due unclear information on HIV testing method, 9 were removed due to replicate study population and 31 were removed for not meeting quality criteria (see above).

**Figure 2:**
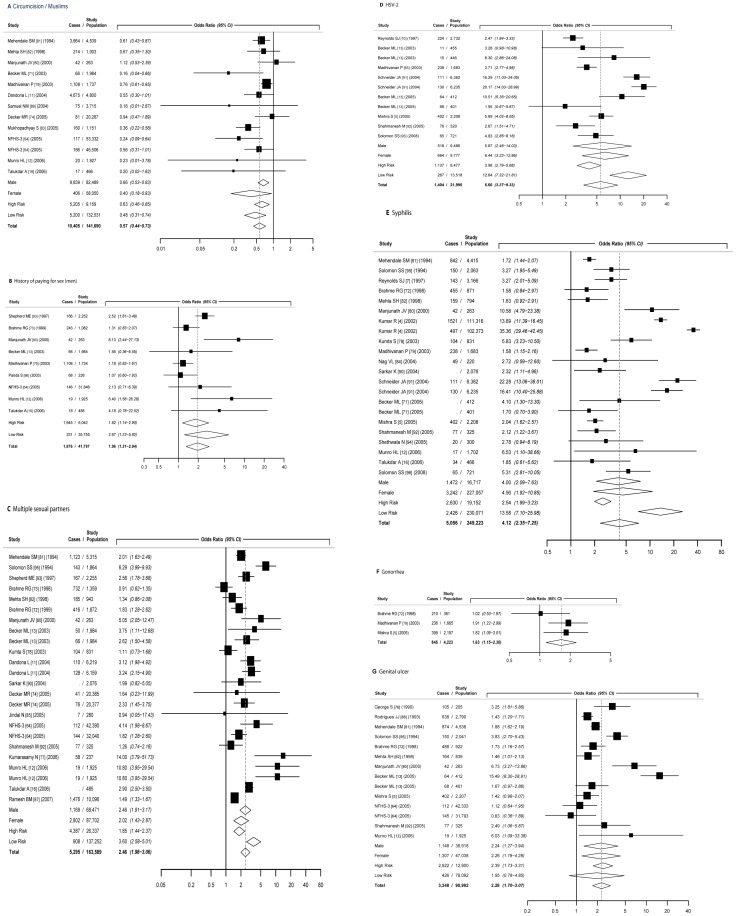
Forest plots from random-effects meta-analysis by risk factor. a. Male circumcision/Muslim religion b. History of paying for sex (men) c. Multiple sexual partners (> = 2 *versus* 0–1) d. HSV-2 e. Syphilis f. Gonorrhea g. History of genital ulcer *Footnotes: i) Study  =  first author, [reference #], year study was conducted. ii) Studies in table (author, publication year [reference #]): Becker, ML 2010* [*71*] *, Becker, ML 2007* [*13*] *, Brahme, R 2006* [*72*] *, Brahme, R 2005* [*73*] *, Dandona, L 2008* [*11*] *, Decker, MR 2009* [*74*] *, Gangakhedkar, RR 1997* [*75*] *, George, S 1997* [*76*] *, Kumar, R 2006* [*4*] *, Kumarasamy, N 2010* [*77*] *, Kumta, S 2010* [*78*] *, Madhivanan, P 2005* [*79*] *, Manjunath, P 2002* [*80*] *, Mehendale, SM 1996* [*81*] *, Mehta, SH 2006* [*82*] *, Mishra, S 2009* [*5*] *, Mukhopadhyay, S 2010* [*83*] *, Munro, HL 2008* [*12*] *, Nag, VL 2009* [*84*] *, Jindal, N 2007* [*85*] *, National Family Health Survey 3 (NFHS-3) 2006* [*64*] *, Panda, S 2005* [*86*] *, Ramesh, BM 2008* [*87*] *, Reynolds, SJ 2003* [*10*] *, Reynolds, SJ 2006* [*7*] *, Rodrigues, JJ 1995* [*88*] *, Samuel, NM 2007* [*89*] *, Sarkar, K 2006* [*90*] *, Schneider, JA 2010* [*91*] *, Shahmanesh, M 2009* [*92*] *, Shepherd, ME 2003* [*93*] *, Shethwala, N 2009* [*94*] *, Solomon, S 1998* [*95*] *, Solomon, S 2010* [*96*] *, Talukdar, A 2007* [*16*] *. iii) For some studies missing cases are shown where effect estimates were available but counts were not calculable from the published study or available from the authors. Some studies may appear more than once due to separate estimates for men and women.*

**Table S1** provides summary information for all 35 study populations included in the final analysis. **Table 1** presents all summary odds ratios for the seven studied risk factors. **Figures 2a to 2g** presents forest plots generated by random-effects meta-analysis for each risk factor including stratification by gender and HIV-risk population. **Table 2** presents estimated study population attributable fractions (PAF).

**Table 1:** Summary table of effect estimates from random effects meta-analysis for seven risk factors.

Exposure	Group	n	Summary OR (95%CI)	I^2^ (%)	X^2^ p	Tau^2^	*p*
**Male circumcision**	Overall	13	0.57 (0.44,0.73)	*44.5*	*0.042*	*0.072*	*0.04*
**or muslim religion**	Males	9	0.66 (0.53,0.83)	*24.4*	*0.227*	*0.026*	
	Females	3	0.4 (0.18,0.93)	*42.2*	*0.177*	*0.226*	
	High-risk group	6	0.63 (0.46,0.85)	*53.8*	*0.055*	*0.066*	
	Gen. pop.	7	0.48 (0.31,0.74)	*33.0*	*0.176*	*0.106*	
	High-risk males group	4	0.72 (0.56,0.92)	*21.3*	*0.283*	*0.015*	
	Gen. males pop.	5	0.56 (0.36,0.87)	*27.7*	*0.237*	*0.068*	
	High-risk females group	1	0.67 (0.35,1.3)	.	.	.	
	Gen. females pop.	2	0.23 (0.1,0.58)	*0.0*	*0.795*	*0.000*	
**Multiple sexual**	Overall	24	2.46 (1.98,3.06)	*84.8*	*<0.0001*	*0.190*	*0.05*
**partners**	Males	11	2.46 (1.91,3.17)	*72.4*	*<0.0001*	*0.110*	
	Females	10	2.02 (1.43,2.87)	*78.0*	*<0.0001*	*0.193*	
	High-risk group	13	1.85 (1.44,2.37)	*85.5*	*<0.0001*	*0.138*	
	Gen. pop.	11	3.6 (2.58,5.01)	*69.0*	*<0.0001*	*0.189*	
	High-risk males group	6	2.2 (1.51,3.2)	*78.6*	*<0.0001*	*0.140*	
	Gen. males pop.	5	2.85 (1.9,4.26)	*68.6*	*0.013*	*0.137*	
	High-risk females group	5	1.33 (1.07,1.64)	*38.0*	*0.168*	*0.022*	
	Gen. females pop.	5	4.05 (2.56,6.41)	*27.9*	*0.235*	*0.076*	
**Paid sex**	Males	9	1.96 (1.31,2.94)	*67.6*	*0.002*	*0.202*	*0.29*
	Females	2	6.46 (4.64,9.01)	*0.0*	*0.659*	*0.000*	**.**
	High-risk males group	6	1.82 (1.14,2.9)	*76.2*	*0.001*	*0.217*	
	Gen. males pop.	3	2.67 (1.23,5.82)	*8.1*	*0.337*	*0.040*	
	High-risk females group	1	6.36 (4.53,8.94)	**.**	**.**	**.**	
	Gen. females pop.	1	9.12 (1.92,43.38)	**.**	**.**	**.**	
**HSV-2**	Overall	11	5.6 (3.37,9.33)	*92.2*	*<0.0001*	*0.634*	*0.92*
	Males	5	5.87 (2.46,14.03)	*93.0*	*<0.0001*	*0.857*	
	Females	5	6.44 (3.22,12.86)	*87.9*	*<0.0001*	*0.513*	
	High-risk group	7	3.98 (2.79,5.68)	*76.9*	*<0.0001*	*0.161*	
	Gen. pop.	4	12.64 (7.32,21.81)	*68.4*	*0.023*	*0.184*	
	High-risk males group	3	3.8 (2.83,5.12)	*14.9*	*0.309*	*0.013*	
	Gen. males pop.	2	15 (6.66,33.79)	*57.4*	*0.126*	*0.222*	
	High-risk females group	3	5.33 (2.75,10.36)	*79.3*	*0.008*	*0.270*	
	Gen. females pop.	2	8.14 (1.72,38.5)	*83.6*	*0.014*	*1.070*	
**Gonorrhea**	Overall	3	1.63 (1.15,2.3)	*23.6*	*0.270*	*0.022*	*0.16*
	Males	1	1.92 (1.23,2.99)	.	.	.	
	Females	2	1.42 (0.81,2.49)	*46.6*	*0.171*	*0.078*	
	High-risk group	3	1.63 (1.15,2.3)	*23.6*	*0.270*	*0.022*	
	High-risk males group	1	1.92 (1.23,2.99)	**.**	**.**	**.**	
	High-risk females group	2	1.42 (0.81,2.49)	*46.6*	*0.171*	*0.078*	
**Syphilis**	Overall	22	4.12 (2.35,7.25)	*97.6*	*<0.0001*	*1.672*	*0.34*
	Males	9	4 (2.09,7.63)	*93.2*	*<0.0001*	*0.821*	
	Females	10	4.56 (1.92,10.85)	*98.2*	*<0.0001*	*1.845*	
	High-risk group	16	2.54 (1.99,3.23)	*70.1*	*<0.0001*	*0.135*	
	Gen. pop.	6	13.58 (7.1,25.98)	*95.1*	*<0.0001*	*0.553*	
	High-risk males group	7	3.04 (1.84,5.01)	*86.2*	*<0.0001*	*0.345*	
	Gen. males pop.	2	15.54 (10,24.14)	*0.0*	*0.325*	*0.000*	
	High-risk females group	7	2.03 (1.69,2.42)	*0.0*	*0.810*	*0.000*	
	Gen. females pop.	3	22.12 (10.82,45.22)	*96.1*	*<0.0001*	*0.371*	
**History of**	Overall	14	2.28 (1.7,3.07)	*86.7*	*<0.0001*	*0.233*	*0.19*
**genital ulcer**	Males	5	2.24 (1.27,3.94)	*77.5*	*0.001*	*0.276*	
	Females	6	2.26 (1.19,4.28)	*90.4*	*<0.0001*	*0.562*	
	High-risk group	10	2.39 (1.73,3.31)	*87.7*	*<0.0001*	*0.210*	
	Gen. pop.	5	1.95 (0.78,4.85)	*86.0*	*<0.0001*	*0.671*	
	High-risk males group	3	2.57 (1.34,4.91)	*83.3*	*0.002*	*0.265*	
	Gen. males pop.	2	1.93 (0.28,13.3)	*76.2*	*0.040*	*1.510*	
	High-risk females group	5	2.61 (1.25,5.45)	*91.8*	*<0.0001*	*0.631*	
	Gen. females pop.	1	1.12 (0.64,1.95)	.	.	.	
							

Footnote:

n =  number of studies; Results of random effects meta-analysis; p =  p-value for Egger's test for publication bias.

**Table 2:** Population attributable fraction estimates.

				Summary	
Exposure	*Group*	n	P_e_^1^	OR	PAF ^2^
**Uncircumcised male/**	*Males*	82,489	78.9%	1.5	29%
**non-muslim religion**	*Females*	58,050	87.1%	2.5	57%
**Multiple sexual**	*Males*	68,471	23.5%	2.5	26%
**partners**	*Females*	87,702	6.7%	2.0	6%
**Paid sex**	*Males*	41,797	7.9%	2.0	7%
	*Females*	2,940	18.2%	6.5	50%
	*High-risk males*	6,042	47.7%	1.8	28%
	*Gen. popl'n males*	35,755	1.2%	2.7	2%
**History of**	*Males*	38,918	10.7%	2.2	12%
**genital ulcer**	*Females*	47,038	11.7%	2.3	13%
**HSV-2**	*Males*	9,486	14.0%	5.9	41%
	*Females*	9,777	24.6%	6.4	57%
**Syphilis**	*Males*	16,717	8.6%	4.0	20%
	*Females*	227,057	2.0%	4.6	7%

Footnotes:

1. Pe  =  prevalence of exposure in study population.

2. PAF  =  Population attributable fraction calculated as: Pe * (OR - 1)/(Pe * (OR - 1)+1).

### Risk factors

#### Male circumcision status/Muslim religion

Among 13 studies, male circumcision status (or Muslim religion) significantly reduced the probability of HIV infection (OR: 0.57; 95%CI: 0.44–0.73) pooled across both genders and risk populations (Figure 2a). Among men, circumcision was associated with an approximately 40% reduction in probability of HIV infection (OR: 0.66; 95%CI: 0.53–0.83). This effect differed between men in high-risk (OR: 0.72; 95%CI: 0.56–0.92) or those in the general population (OR:0.56; 95%CI: 0.36–0.87) but not significantly. Among women overall, Muslim religion was also significantly associated with reduced probability of HIV infection (OR: 0.40; 95%CI: 0.18–0.93). There were an insufficient number of studies to look at differences between high- and low-risk populations among women. In our study population one of the largest attributable fractions for women was non-Muslim religion. Assuming an indirect causal association (due to male circumcision), approximately 60% of all HIV infections among women in our study population were attributed to non-Muslim religion (Table 2).

#### Sexual Behaviour

Having two or more lifetime sexual partners was significantly associated with HIV positivity (OR: 2.46; 95%CI: 1.98–3.06) pooled across both genders and risk populations (Figure 2c). Among men, having two or more sexual partners was significantly associated with an increase in probability of HIV infection (OR: 2.46; 95%CI: 1.91–3.17). This effect size was similar for high-HIV (OR = 2.20; 95%CI: 1.51–3.20) and general population men (OR: 2.85; 95%CI: 1.90–4.26). Among women, reporting multiple sexual partners was also significantly associated with increased probability of HIV infection (OR: 2.02; 95%CI: 1.42–2.87) pooled across risk populations. The association between multiple sex partners and HIV was also significantly stronger among women from the general population (OR: 4.05; 95%CI: 2.56–6.41) than women from high-HIV risk populations (OR: 1.33; 95%CI: 1.07–1.64).

Among men, paying for sex was significantly associated with HIV positivity (OR: 1.96; 95%CI: 1.31–2.94) pooled across both risk populations **(** **Figure 2b** **)**. This effect was slightly different for high-risk and general population men (OR = 1.82; 95%CI: 1.14–2.90, OR: 2.67; 95%CI: 1.23–5.82, respectively). Only two study estimates were available to estimate association between being paid for sex and HIV status among women, both suggesting a very high probability of infection (OR_women_ = 6.46; 95%CI: 4.64–9.01) (data not shown).

#### Sexually transmitted infections

Among 11 studies, HSV-2 infection increased the probability of HIV infection (OR: 5.60; 95%CI: 3.37–9.32) pooled across both genders and risk populations (Figure 2d). Among men, HSV-2 infection was associated with an approximately five times increase in probability of HIV infection (OR: 5.87; 95%CI: 2.46–14.03). Among women overall, HSV-2 status was also significantly associated with increased probability of HIV infection (OR: 6.44; 95%CI: 3.22–12.86). These patterns were also observed among high- and low HIV risk men and women. Associations between HSV-2 and HIV were stronger among general population study groups than high-risk.

Syphilis infection was associated with a quadrupling of likelihood of HIV infection (OR: 4.12; 95%CI: 2.34–7.25) pooled across both genders and risk populations **(** **Figure 2e** **)**. These findings were true for both genders and risk populations. The association between syphilis and HIV was much stronger among general-population groups and this pattern was seen for both genders.

Only three studies were included that reported on the association between biologically confirmed history of gonorrhoea infection and HIV and these were all in high-risk populations **(** **Figure 2f** **)**. Gonorrhoea was significantly associated with increased probability of HIV infection (OR: 1.63; 95%CI: 1.15–2.30) and this effect size was similar for both men and women.

Self-reported history of genital ulcer was significantly associated with increased probability of HIV infection (OR: 2.28; 95%CI: 1.70–3.07) pooled across both genders and risk populations **(** **Figure 2g** **)**. This effect magnitude was also observed for each sex. History of genital ulcer was more strongly associated with HIV among high-risk groups however this difference was not significant. Among women, all included studies (n = 6) were in high-risk populations except for one.

### Publication bias, heterogeneity, influence of individual studies and effect modification

Evidence of significant publication bias (protective effect) was only observed for male circumcision status/female Muslim religion (p_Egger's test_ = 0.04) (**Figure S1**). Heterogeneity in effect estimates (I^2^) are reported in **table 1**. Significant heterogeneity was observed but declined upon stratification by gender and HIV-risk populations and sensitivity analysis suggest that study results were robust for each of the seven risk factors examined (**Figure S2)**.

The effects of study characteristics as analysed by random-effects meta-regression are presented in **Table S2**. Overall summary effect measures for all six risk factors (too few studies were available to conduct meta regression for gonorrhea) were not associated with gender, study year, study design or state. The associations between HSV-2, syphilis and multiple partnerships with HIV status were stronger among low-HIV risk groups and HIV test method (western blot *versus* other) was associated with larger effect size for multiple sex partners.

## Discussion

We have previously estimated that HIV transmission probability during partnership between discordant couples in India is low (30% to 46%) [28]. This suggests that co-factors for HIV transmission play an important role in the size of the Indian epidemic. The results of our meta-analysis are in line with evidence from other parts of the world that lack of male circumcision, use of commercial sex work, having multiple sexual partners and a history of STI all increase probability of HIV infection [29], [30], [31].

### Circumcision

Male circumcision has been shown in randomized controlled trials in the African continent to significantly reduce risk of HIV infection in men by approximately half and indirectly, through their own risk reduction, in their partners and wives [32], [33], [34], [35], [36]. In India male circumcision is largely restricted to Muslims and a reasonable estimate of prevalence of male circumcision would be approximately 12% (proportion of the Indian population that is Muslim). While no trials of circumcision have been carried out in India, observational studies have strongly supported these previous African findings [6], [35]. We found similar evidence for the Indian setting with a summary OR suggesting a halving of probability of HIV positivity. The biological basis for this effect has been discussed in the literature [37] and there is now little debate that its effect is due to biological action rather than behaviour associated with being Muslim. This would explain the general consistency of the effect across risk groups and genders and the results of randomized controlled trials. While we did not examine non-HIV STIs as outcomes, one Indian study suggested that the protective effect of male circumcision against HIV infection was specific for HIV and did not extend to other STIs such at syphilis and gonorrhoea [16]. This finding is also consistent with data from African settings [38]. While studies have reported lower cervical cancer prevalence among Muslim women in India [39], a recent retrospective cohort study of 524 women in rural eastern India suggests that Muslim women were no less likely than Hindu women to be infected with human papilloma virus 16/18 or to develop abnormal cervical cytology [40].

Circumcision was expected to be protective against HIV infection among men and not surprisingly among women as well. Muslim religious status among Indian women had a larger (though not significantly so) protective effect against HIV than in men and was the largest summary effect size among women in our study. This may be due to a combination of reduced exposure to HIV through their Muslim male partners and to behavioural reductions in risk. Among high risk women, which included FSWs, this protective effect could represent sorting of clients based on religion or participation in sex work based on location (Muslim FSWs being more likely to work in Muslim neighbourhoods and have Muslim clients). Among women in our study population, the single largest contributor to HIV risk was non-Muslim religion. This supports the prevailing theory that male sexual activity outside of regular partnerships is a key driver of the HIV epidemic in India.

### Sexually transmitted infections

Sexually transmitted infections are a risk factor for HIV acquisition but can also increase onward HIV transmission and are therefore hypothesized to play an important role in HIV transmission dynamics in India [9], [10], [13], [41]. STIs are thought to exert their effects on HIV transmission via genital lesions however even in the absence of such lesions STIs can increase the efficiency of HIV transmission [5]. STIs may act as stronger risk factors for HIV transmission in developing countries like India because of socio-economic barriers to treatment.

HIV infection among men was most strongly associated with HSV-2 infection. Among men in our study population, HSV-2 had the largest summary association measure with HIV infection and was estimated to be causally associated with almost half of their HIV infections. In women HSV-2 positivity was the strongest risk factor examined for HIV infection for which there were a sizeable number of study estimates. There is a high degree of variation in HSV-2 prevalence estimates in India, particularly in high risk groups. HSV-2 prevalence has been reported between 1.0% and 18.9% from general population-based surveys,[6], [10], [13], [18], [42], [43], [44], [45], [46] between 9.7% and 83% from STD clinics,[6], [10], [47] and between 2.0% and 79.0% from high-risk group surveys[48], [49], [50], [51]. In addition to causing significant morbidity, HSV-2 is a leading cause (∼50%) of genital ulcers in developing countries[52]. Ulcerative STIs have been associated with increased risk of HIV infection [5], [53], [54], [55]. While the association between these STIs and HIV infection is strong, one cannot rule out reverse causality (particularly when studies reported a test result indicating “ever” infection with STI, such as VDRL test for syphilis). HIV weakens the immune system and therefore makes one more susceptible to infections including all STIs [56]. Furthermore, subjects with HSV-2 and HIV may share similar sexual behaviours, position in a sexual network and have HIV-positive partners who are more likely to transmit HSV-2 at the same time as HIV. Despite a large amount of epidemiological evidence suggesting a key causative role, eight of nine randomized trials of treatment of HSV-2 infection to reduce HIV incidence have found insignificant results. However, several issues around trial design and conduct have been argued to be important modifiers of STI treatment effect on HIV incidence [57].

The prevalence of syphilis in India is unknown but estimates from antenatal clinic attendees suggest a prevalence around 1.5%[5], [58]; estimates for syphilis among FSWs are closer to 20% [3], [5], [59]. There has been debate in the literature about the role of STIs in HIV transmission and particularly whether viral *versus* bacterial STIs play a more significant role and the difficulty in teasing apart independent effects [21], [22]. Our results suggest that, based on strength of effects, both viral (HSV-2) and bacterial STIs (syphilis and gonorrhoea) have a similar association with HIV prevalence overall. There was some evidence that the overall effects of both syphilis and gonnorhea, for both sexes, were weaker when compared to HSV-2. This pattern was consistent for men when studies were stratified by HIV-risk populations but not women. The stronger association with HIV for HSV-2 compared to syphilis has been noted previously[60], [61]. We noted that few or no included studies examined the associations between gonorrhea or genital ulcer and HIV in general population women and that this represents an important gap in the literature.

Meta-regression results suggested that the association of syphilis with HIV was stronger among low-HIV-risk population (p<0.001). This was mostly due to syphilis being substantially more strongly associated with HIV status among low-risk women (antenatal clinic attendees) than high-HIV-risk women. This likely represents the effect of markedly different background prevalence of exposure. Among high-HIV-risk groups, syphilis was more prevalent generally in both cases and controls whereas in low-HIV-risk groups syphilis was generally rare (as was HIV infection) but still associated with HIV leading to a stronger association.

We expected genital ulcer to be more strongly and consistently associated with male risk of HIV. This would have been congruent with other epidemiologic findings [31]. Genital ulcer was reported as a self-reported history thus could be susceptible to social desirability bias with women being more prone to underreporting than men. Furthermore, genital ulcers in women are less likely to be diagnosed as they are not easily visible as in men and are generally painless [5], [62], [63]. We did not see a difference in the relationship between HIV and genital ulcer for men and women. The difference in strength of association between genders for biologically confirmed STIs such as syphilis and HSV-2 (which should not be susceptible to social desirability bias) was also not evident.

### Sexual behaviour: Paid sex & multiple sexual partners

It is suggested that the Indian HIV epidemic is driven by heterosexual sex and particularly by male use of commercial sex work [3], [8], [19]. The results of this meta-analysis suggest that paying for sex was indeed associated with HIV infection among men however not as strongly other risk factors. The summary estimate from nine studies suggests an approximate doubling of risk which is lower than the effect sizes for the other six risk factors. This pattern held even when stratifying studies by HIV-risk population. The literature on risk of HIV among men paying for sex in sub-Saharan Africa suggests an effect size of similar magnitude [31].

Compared to paying for sex, all STI exposures and reporting multiple sexual partners were more strongly associated with HIV infection and this was true even when restricted to men from high-risk populations. We have previously estimated that a sizable minority (10 to 20%) of HIV infections in married couples in India are introduced by the female partner [28]. These results appear to agree roughly with data from a nationally representative sexual behaviour survey conducted in 2006 in the general population where 5.2% of married men reported a non-regular partner during the past year compared with 1.7% of married women [64]. However the relationship with multiple sex partners may have been stronger as non-regular sexual partnership was likely underreported. We have shown that among HIV-positive partners in HIV-discordant married couples in India, 75% of husbands and 88% of wives reported one lifetime sexual partner [28] and in a separate community household survey, prevalence of HSV-2 and syphilis was 2% and 1%, respectively, among women reporting never having had sex [65]. Self reported paid sex may similarly be subject to social desirability bias and therefore carry a greater amount of measurement error.

### Study limitations

Our study had several limitations. Firstly, only 35 studies were included in the final analysis of seven risk factors with two stratification levels. This limited our statistical power to find weaker associations and the extent to which we could examine other factors thought to influence the relationship between our seven exposures of interest and HIV status through stratification or meta-regression. The range of study years for each risk factor was limited prohibiting robust analysis of trends in effect sizes over time. Several studies did not provide sex-specific effect estimates and this limited our ability to explore effects between genders. While we received sex-stratified tables from several authors, we were unable to get these data for all studies.

The HIV epidemic in India is geographically heterogeneous with 75% of reported HIV cases in four large southern states that house 30% of the country's population [3], [58]. Reasons for this geographic variation are still unclear and could not be explored effectively with our analysis. Geography was not found to be associated with study effects for each of the seven risk factors examined. However there was limited variation in the study estimates as only nine states were represented in our dataset and 25 of the 35 included studies were conducted in one of the large southern states.

There was a wide range (I^2^ = 0.0–98.2% – among summary effects for each gender) of heterogeneity in effect of exposures across studies. However the I^2^ measure does not work well for cohorts and in prevalence studies generally yields higher values. Some of this heterogeneity may be due to smaller studies which were included or studies in which few exposed cases or controls were observed. Random effects methods were used to help account for this higher level of heterogeneity.

We followed the MOOSE group [23] and Greenland et. al. [24] recommendations for conducting meta-regression on study characteristics that may have contributed to variation in effects. We chose to use a random-effects model partly because of the between-study variation in effect estimates however it has been argued that reliance on random effects methods accommodates important variation, which should be explained, rather than adjusted for [24]. We found that low HIV risk group was associated with higher effect sizes for HSV-2, syphilis and multiple sexual partners. This may be due to these exposures more specifically identifying HIV infection in a lower prevalence background. HIV test method (Western blot) was associated with larger effect sizes, however six out of nine studies that employed Western blot for HIV test method were among high HIV-risk populations.

Measurement error is an important issue in any epidemiologic study. The use of face-to-face interviews to measure sexual behaviour could lead to misreporting due to social desirability bias. We found a range of HIV prevalence estimates (0.3 to 7.3%) in populations that were categorized as being from the general population (and assumed to be at low-risk for HIV infection). This suggests that some individuals in the general population samples were not at low risk of HIV and perhaps were more accurately categorized as high-risk. Under-reporting by women would have biased the summary effect estimate of multiple partnerships towards the null assuming that women with and without HIV were equally likely to under-report non-regular partnerships. In general, men have been shown to over report numbers of sex partners in sexual behaviour surveys (although this is not always the case [66], [67]) and this tendency would have increased the effect estimate if those over reporting multiple sex partners were more likely to be HIV infected. We attempted to minimize measurement error in the outcome by assessing whether each study had reported determination of HIV status by WHO guidelines for HIV testing in a developing country setting [68]. Similarly specific STIs required biological test result rather than self-reported history of diagnosis. We were unable to explore the potential effect of different methods of STI measurement in meta-regression due to the number of methods used and multiple methods used in single studies. This could have contributed to measurement error in our study.

### Implications

The results of our systematic review and meta-analysis suggest that sexual behaviour outside of regular partnerships is a key drivier of the HIV epidemic in India. The robust role of multiple partnerships was emphasized by the observation of equally strong effect size for both genders and across HIV–risk populations. Co-factors for HIV transmission likely play an important role in the size of the Indian epidemic given the relatively low probability of HIV transmission [28], [29], [69]. Risk factors for men and women differed in their strengths however the strength of association of STIs with HIV, particularly HSV-2 was notable. The risk factors examined in our analysis should remain targets of HIV prevention programs even in the context of a heterogeneous HIV epidemic [70].

## Supporting Information

Figure S1 Funnel plots for publication bias (p-value for Egger's test). a) Male circumcision or Muslim religion b) Multiple sexual partners c) Paid for sex (men) d) Genital ulcer e) Syphilis f) HSV-2 *Footnote: P =  p-value for Egger's test for publication bias.*
(TIFF)

Figure S2 Influence plots for summary odds ratio by risk factor. a) Male circumcision/female religion status b) History of paying for sex (men) c) Multiple sex partners d) HSV-2 e) Syphilis f) Genital ulcer *Footnote: Each estimate represents the estimated summary odds ratio after the removal of the given study est*
(PPT)

Table S1 Summary of study population characteristics. *Footnote: Circ./Muslim = male circumcision or Muslim religion; HSV-2 = Herpes Simplex Virus 2; State: KN = Karnataka, MH = Maharashtra, AP = Andhra Pradesh, S.India =  South India; Population type: STI = Sexually transmitted infection clinic attendees; General = general population survey, FSW = female sex worker, MSM = Men who have sex with men, ANC = antenatal clinic attendees, IDU = injecting drug users; Design: CS = cross-sectional, CHRT = cohort, CCTRL = Case-control; HIV risk group: H = high, L = low (see methods).*
(PDF)

Table S2 Results of random effects meta-regression for six study characteristics. *Footnotes: 1. State =  Four large south Indian states versus all others. 2. F-test for categorical variables*
(DOC)
