# Supplementary figures and images for: A Systematic Review and Meta-Analysis of Risk Factors for Sexual Transmission of HIV in India
Source: PLoS One. 2012 Aug 28;7(8):e44094. doi: 10.1371/journal.pone.0044094 (PMC3429412; doi:10.1371/journal.pone.0044094)

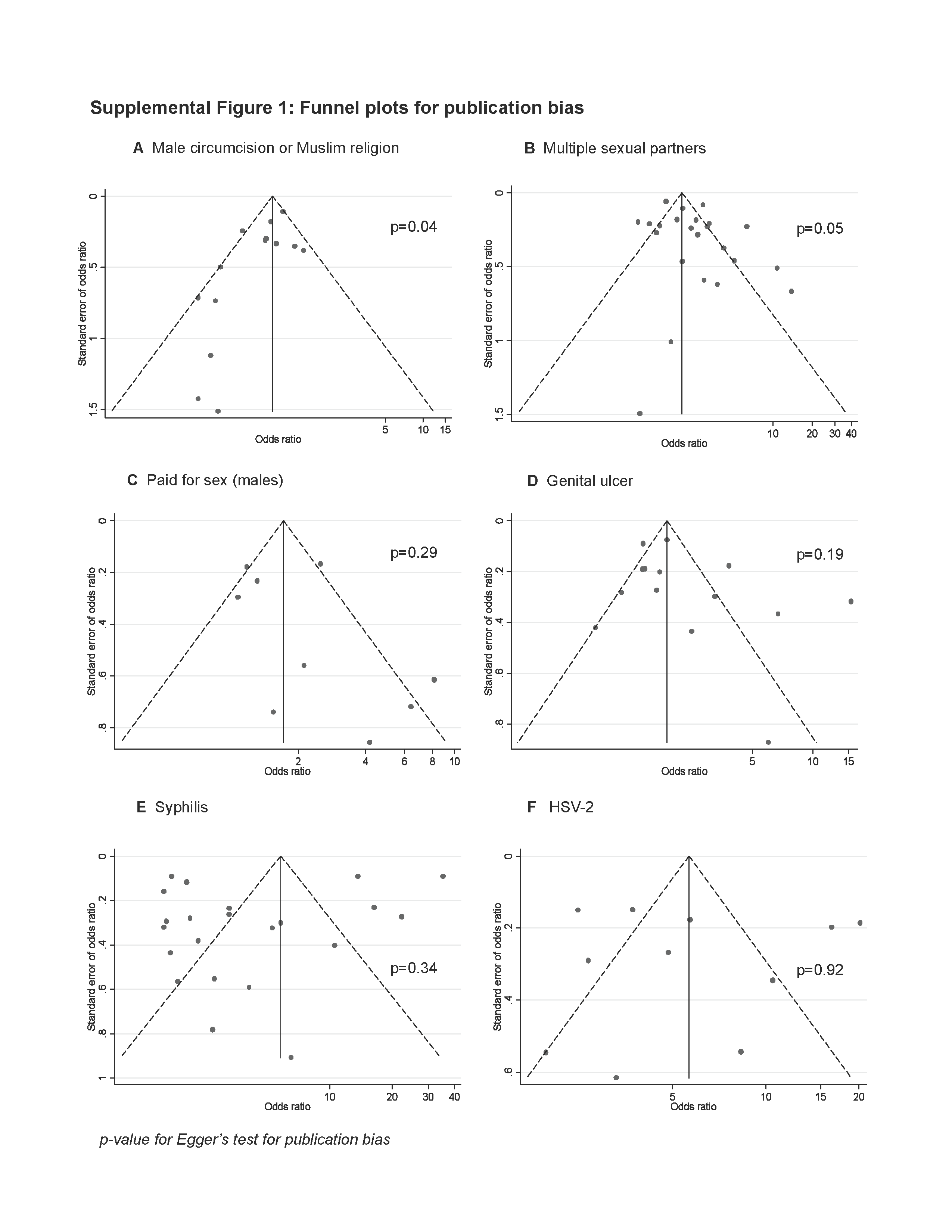

Supplement: Figure S1 — Funnel plots for publication bias (p-value for Egger's test). a) Male circumcision or Muslim religion b) Multiple sexual partners c) Paid for sex (men) d) Genital ulcer e) Syphilis f) HSV-2 Footnote: P = p-value for Egger's test for publication bias. (TIFF) [file pone.0044094.s001.tiff]
