# Supplementary material for: A Systematic Review and Meta-Analysis of Risk Factors for Sexual Transmission of HIV in India
Source: PLoS One. 2012 Aug 28;7(8):e44094. doi: 10.1371/journal.pone.0044094 (PMC3429412; doi:10.1371/journal.pone.0044094)
Supplement: Figure S2 — Influence plots for summary odds ratio by risk factor. a) Male circumcision/female religion status b) History of paying for sex (men) c) Multiple sex partners d) HSV-2 e) Syphilis f) Genital ulcer Footnote: Each estimate represents the estimated summary odds ratio after the removal of the given study est (PPT) [file pone.0044094.s002.ppt]

## Slide 1
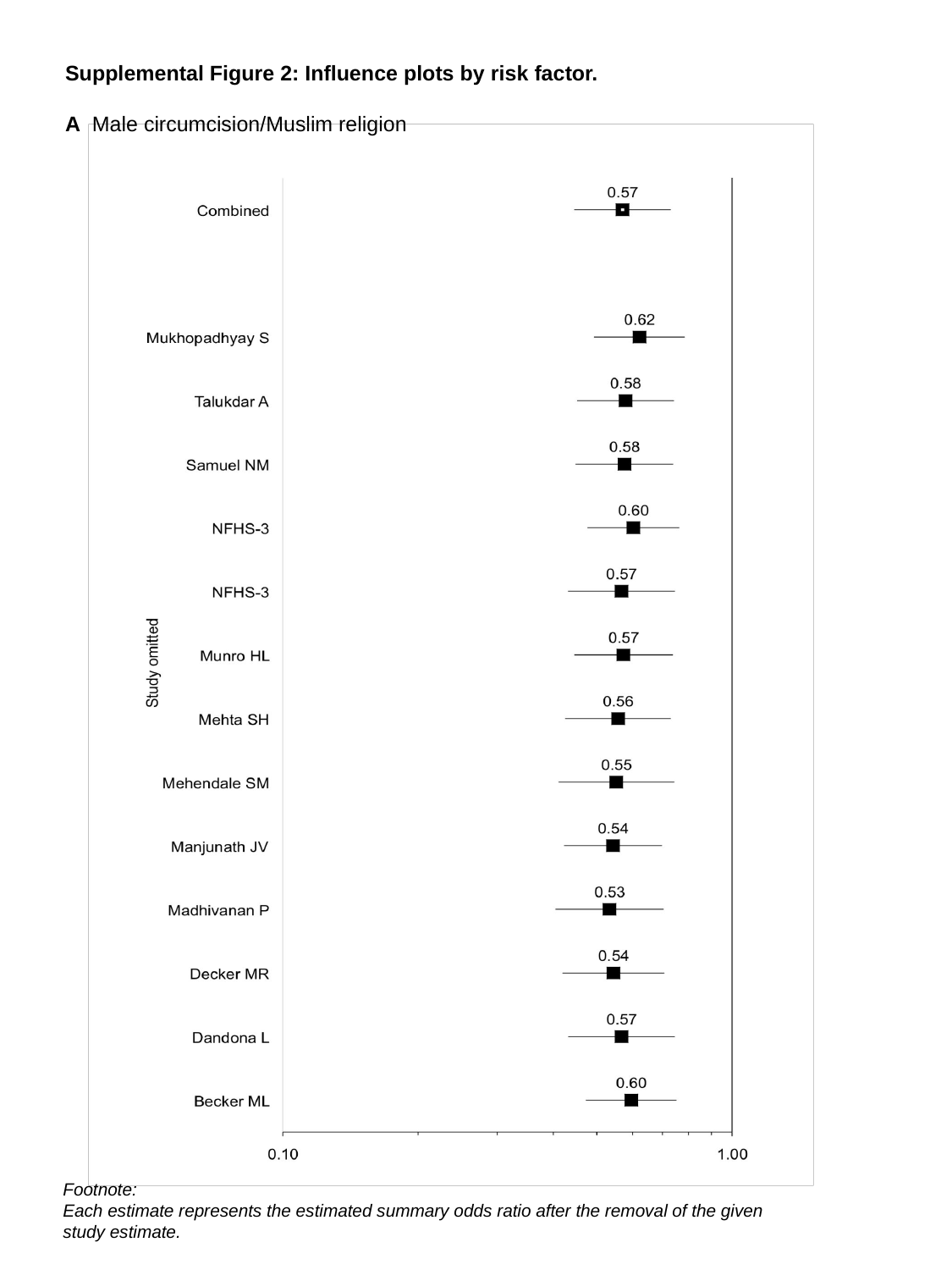

Supplemental Figure 2: Influence plots by risk factor.
A Male circumcision/Muslim religion
Footnote:
Each estimate represents the estimated summary odds ratio after the removal of the given study estimate.

## Slide 2
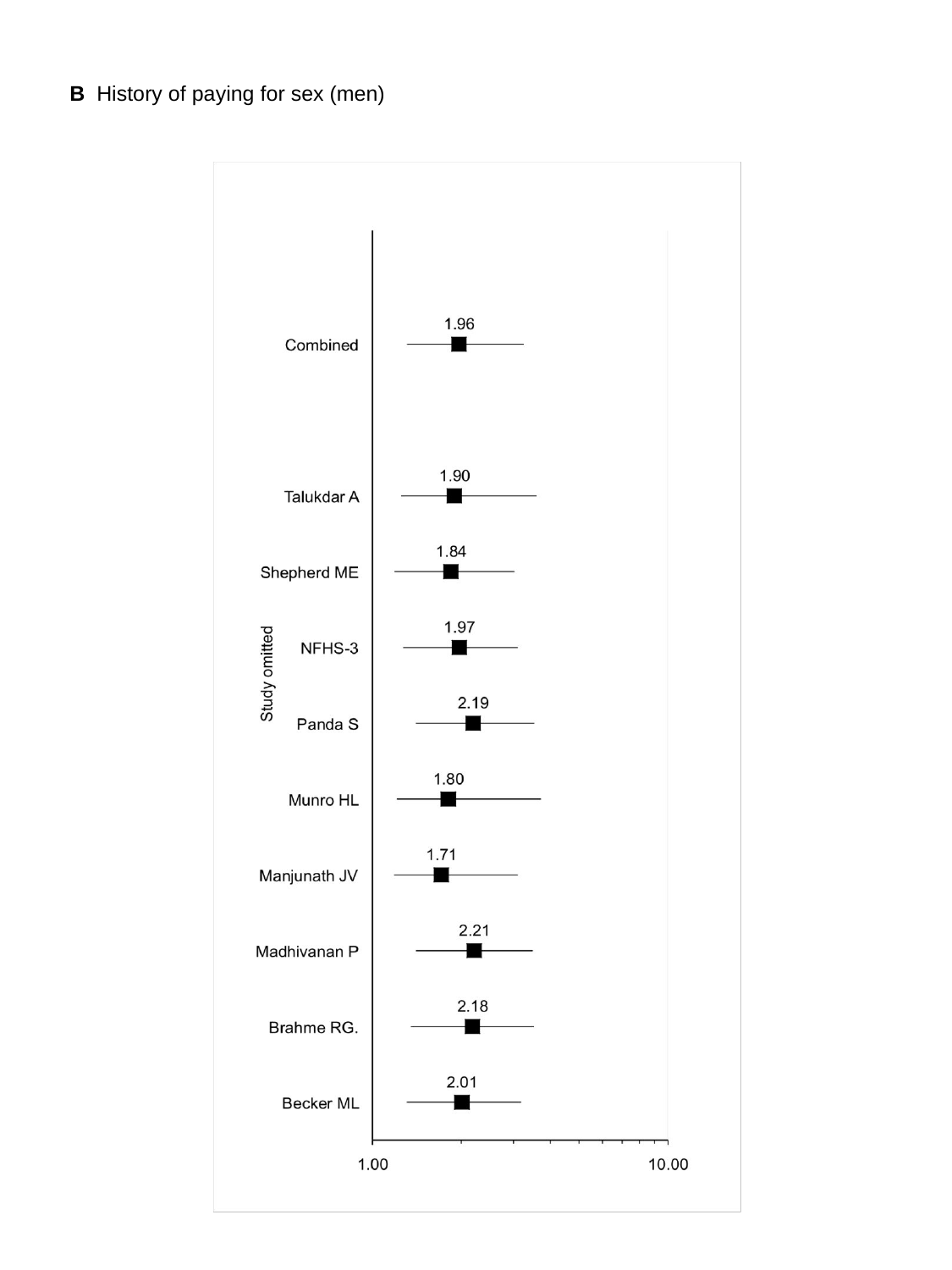

B History of paying for sex (men)

## Slide 3
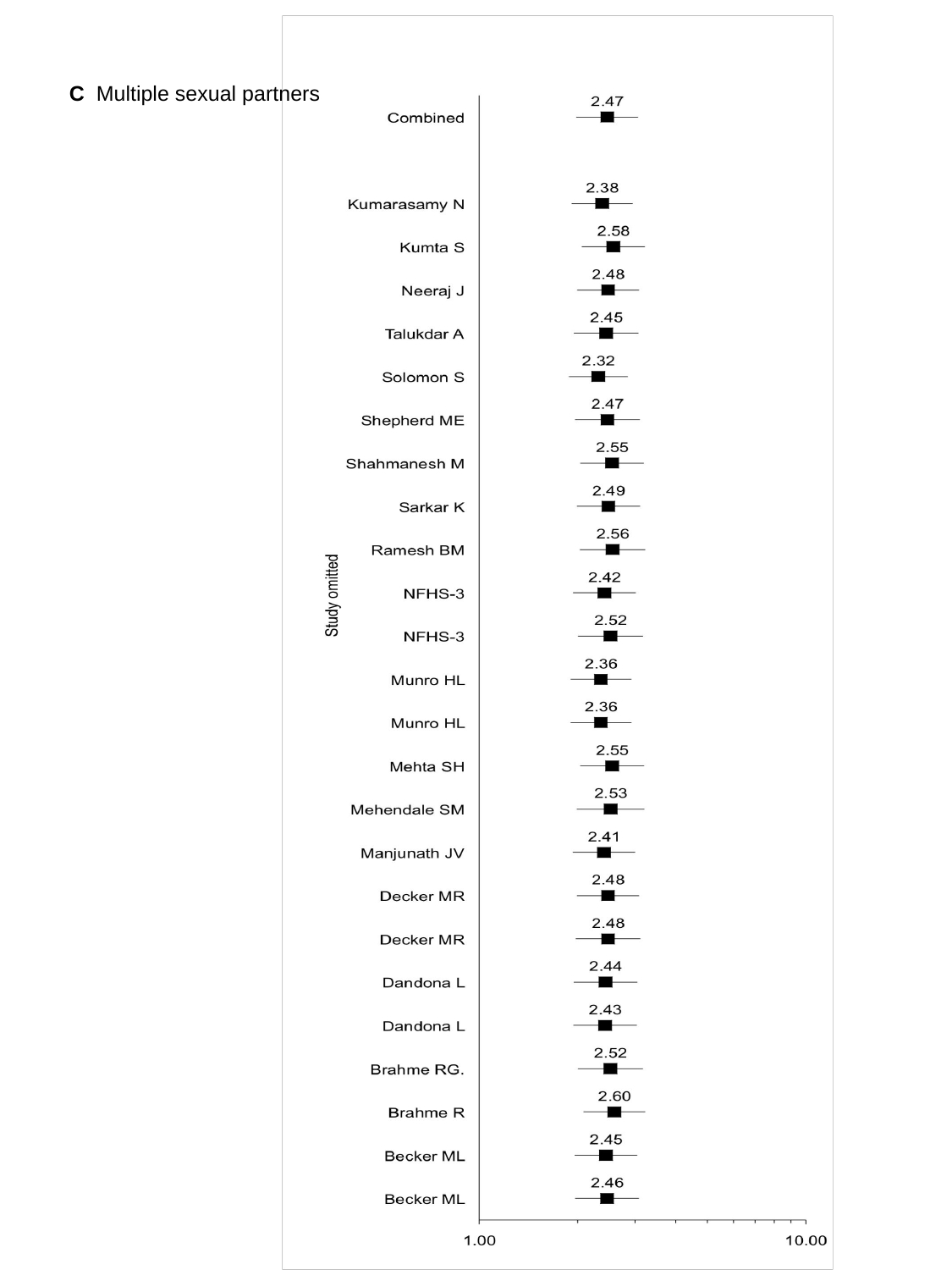

C Multiple sexual partners

## Slide 4
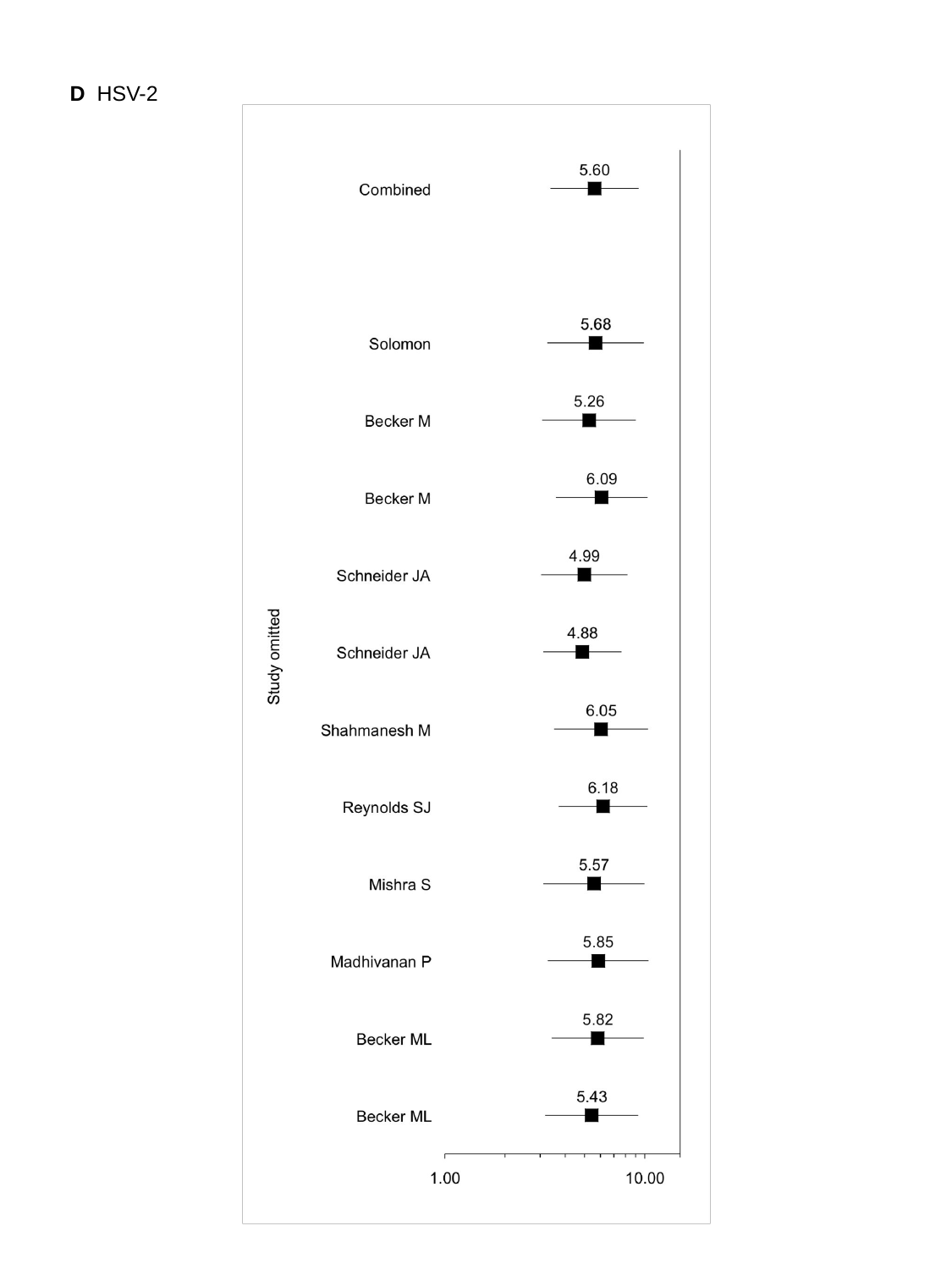

D HSV-2

## Slide 5
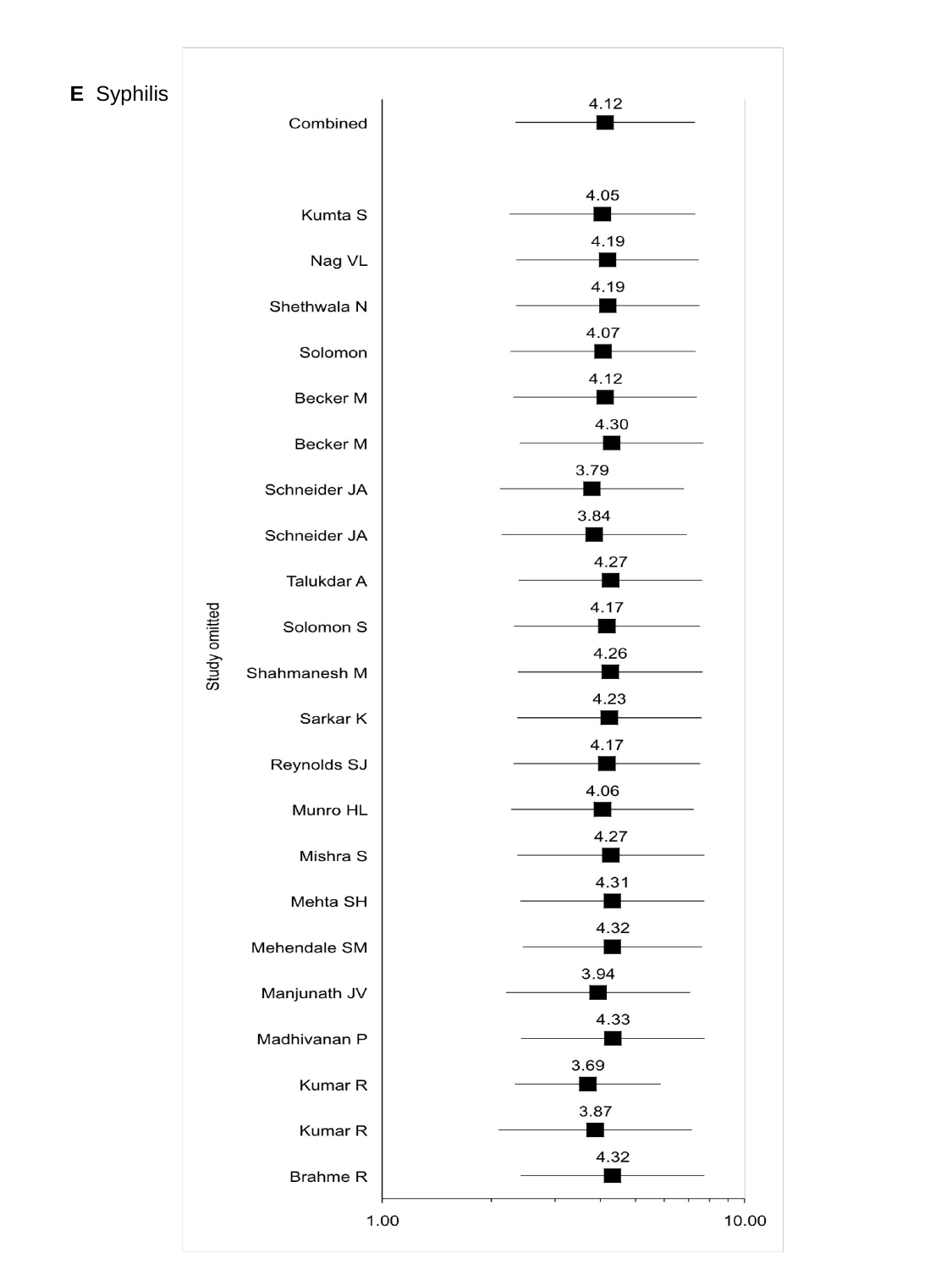

E Syphilis

## Slide 6
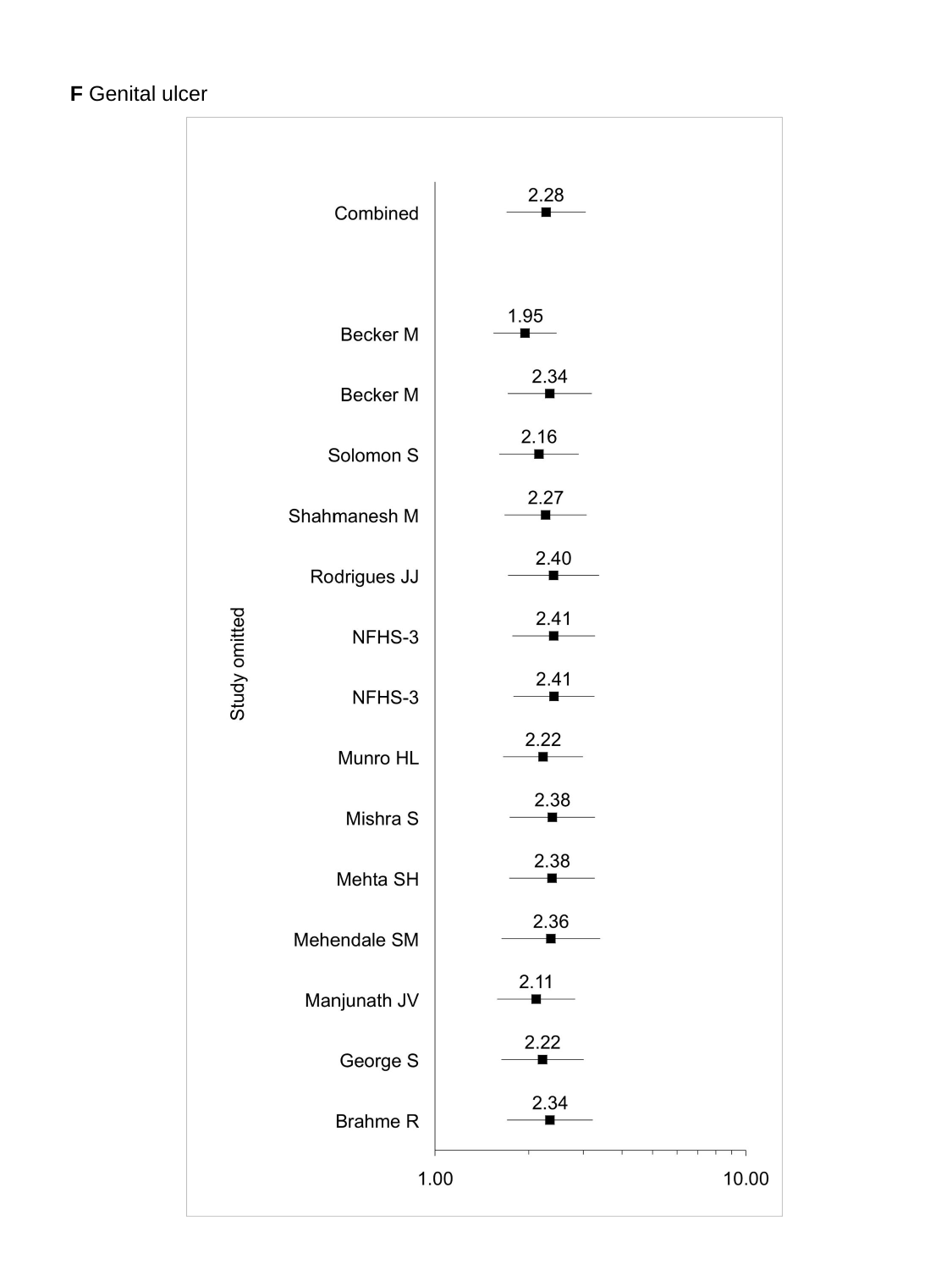

F Genital ulcer
