# Supplementary material for: A Systematic Review and Meta-Analysis of Risk Factors for Sexual Transmission of HIV in India
Source: PLoS One. 2012 Aug 28;7(8):e44094. doi: 10.1371/journal.pone.0044094 (PMC3429412; doi:10.1371/journal.pone.0044094)
Supplement: Table S1 — Summary of study population characteristics. Footnote: Circ./Muslim = male circumcision or Muslim religion; HSV-2 = Herpes Simplex Virus 2; State: KN = Karnataka, MH = Maharashtra, AP = Andhra Pradesh, S.India = South India; Population type: STI = Sexually transmitted infection clinic attendees; General = general population survey, FSW = female sex worker, MSM = Men who have sex with men, ANC = antenatal clinic attendees, IDU = injecting drug users; Design: CS = cross-sectional, CHRT = cohort, CCTRL = Case-control; HIV risk group: H = high, L = low (see methods). (PDF) [file pone.0044094.s003.pdf]

Supplemental Table 1: Summary of study population characteristics.

Study [Ref. number] Year of pub.	Time period of study	Exposures measured	Exposure measurement method	HIV method	State	Population type	Design	*Risk group*	*HIV inc or prev*	Sex specific estimates available?	Prev. of exposure	Overall prev. of HIV	Study sample size	Notes on exposure measurement
**Becker ML [71] 2010**	2004-2006	GUD	Self-report	2 ELISAs	KN	STI	CS	H	P	Y	34.4%	7.9%	813	Current GUD
		HSV-2	multiplex PCR if GUD; IgG ELISA								7.7%			Ever infection
		Syphilis	RPR + TPHA								5.3%			Active infection
**Becker ML [13] 2007**	2003	Circ./Muslim	Self-report	2 ELISAs	KN	General	CS	L	P	Y	15.9%	2.9%	4,008	Religion
		HSV-2	IgG ELISA								18.9%			Ever infection
		Sexual partners	Self-report								7.3%			>1 in last 3 months, 12 months & lifetime
		Paid for sex	Self-report								2.0%			Ever paid or received money for sex
**Brahme R [72] 2006**	1993-2002	Gonorrhea	Gram stain + culture	ELISA + Rapid test	MH	FSW	CS	H	P	Y	11.4%	54.0%	1,359	Active infection
		GUD	physical exam; Self-report								13.1%			Diagnosis of genital ulcer; Hx of genital ulcers
		Sexual partners	Self-report											Lifetime number of partners (1-100, 101-1000, >1000)
		Syphilis	RPR + TPHA								4.9%			
**Brahme RG. [73] 2005**	1998-2000	Sexual partners	Self-report	ELISA + Rapid test	MH	STI	CS	H	P	Y	85.5%	22.2%	1,872	Lifetime number of partners (1, 2-3, 4-10, >10)
		Paid for sex									9.6%			
**Dandona L [11] 2008**	2004-2005	Circ./Muslim	Self-report	1 ELISA or Rapid test	AP	General	CS	L	P	Y	84.0%	1.9%	12,378	Circumcision
		Sexual partners	Self-report								29.7%			Women: >1 in lifetime; men: either >1 in lifetime, or having visited a sex worker
**Decker MR [74] 2009**	2006	Circ./Muslim	Self-report	2 ELISAs	INDIA	General	CS	L	P	Y	11.7%	0.3%	40,762	Religion & circumcision
		Sexual partners	Self-report								9.9%			>1 in lifetime
**Gangakhedkar RR [75] 1997**	1993-1996	Paid for sex	Self-report	ELISA, Rapid, + WB	MH	STI	CS	H	P	Y	57.3%	34.4%	916	Ever receiving money for sex, or identified occupation as SW or former SW
**George S [76] 1997**	1986-1993	GUD	Self-report	ELISA + WB	S. INDIA	STI	CCTRL	H	P	N	40.0%	50.0%	210	Hx of GU
		Paid for sex	Self-report								81.0%			Sexual exposure to a CSW
**Kumar R [4] 2006**	2000-2004	Syphilis	VDRL	2 ELISAs	INDIA	ANC	CS	L	P	Y	1.6%	0.9%	213,689	VDRL reactive
**Kumarasamy N [77] 2010**	2006-2008	Sexual partners	Self-report	Rapid + WB	AP	STI	CCTRL	H	I	N	6.3%	29.5%	237	>1 in past month
**Kumta S [78] 2010**	2003-2004	Sexual partners	Self-report	3 Rapid tests	MH	MSM	CS	H	P	Y	41.0%	12.5%	831	<5, >=5 partners in past 6 motnhs
		Syphilis	VDRL					H			6.5%			VDRL reactive
**Madhivanan P [79] 2005**	2002-2003	Circ./Muslim	Self-report	ELISA + WB	MH	FSW clients	CS	H	P	Y	24.6%	14.0%	1,741	Religion
		Gonorrhea	PCR; Gram stain + culture if genital ulcer								7.3%			Gonococcal urethritis if any are +
		HSV-2	physical exam; IgG ELISA; multiplex PCR if GUD								40.9%			PCR, IgG ELISA and clinical diagnosis used to determine incident, chronic or recurrent infection
		Paid for sex	Self-report								91.7%			Sex with an FSW in last 3 months, ever
		Syphilis	physical exam; VDRL + TPHA; multiplex PCR if GUD								20.4%			Clinical diagnosis, PCR, VDRL and TPHA all used to determine primary, secondary, or latent/treated
**Manjunath JV [80] 2002**	1999-2001	Circ./Muslim	Self-report	ELISA + Rapid test	MH	Truckers	CS	H	P	Y	24.3%	16.0%	263	Religion + circumcision
		GUD	Self-report; physical exam								31.9%			Hx in last 5 years; clinical diagnosis of ulcerative STD
		Sexual partners	Self-report								59.3%			>1 (time period not specified); median number of sex partners in past year given
		Paid for sex	Self-report								66.5%			Sex with CSW (time period not specified)
		Syphilis	VDRL								13.3%			VDRL reactive
**Mehendale SM [81] 1996**	1993-1995	Circ./Muslim	Self-report	ELISA, Rapid, + WB	MH	STI	CHRT	H	P	Y	93.6%	21.2%	5,321	Circumcised
		GUD	physical exam + rapid lab								50.2%			Presenting genital ulcer; Hx of genital ulcer
		Sexual partners	Self-report								84.0%			lifetime partners (1, 2-9, 1-99, 100-999, >1000)
		Syphilis	physical exam + rapid lab								17.0%			VDRL reactive
**Mehta SH [82] 2006**	1993-2002	Circ./Muslim	Self-report	ELISA + Rapid test	MH	STI	CS	H	P	Y	7.0%	21.0%	1,020	
		GUD	Self-report; phys. Exam. + Gram stain, wet mount, dark ground micr.								25.0%			Hx of genital ulcer; genital ulcer on exam
		Sexual partners	Self-report								13.8%			Lifetime partners (1, 2-9, >10)
		Syphilis	VDRL/RPR + TPHA								8.1%			VDRL or RPR +, & TPHA +
**Mishra S [5] 2009**	2004-2006	gonorrhea	PCR	ELISA + Rapid test	KN	FSW	CS	H	P	Y	3.6%	18.2%	2,208	PCR +
		GUD	Self-report								7.7%			Genital ulcer within last 12 months
		HSV-2	2 ELISAs (IgG)								67.8%			
		Syphilis	RPR + TPHA								25.3%			Active if both +; lifetime if TPHA +
**Mukhopadhyay S [83] 2010**	2005-2006	Circ./Muslim	Self-report	3 Rapid tests	WB	STI	CS	H	P	N	26.3%	13.9%	1,151	Religion
**Munro HL [12] 2008**	2005-2006	Circ./Muslim	Self-report	2 ELISA tests/ELISA + WB	KN	General	CS	L	P	Y	10.1%	0.8%	4,190	Religion + circumcision
		GUD	Self-report								1.5%			Ever Hx of genital ulcer
		Sexual partners	Self-report								4.3%			Lifetime partners (0, 1, >1)
		Paid for sex	Self-report								2.2%			Paid for or received money for sex

Study [Ref. number] Year of pub.	Time period of study	Exposures measured	Exposure measurement method	HIV method	State	Population type	Design	*Risk group*	*HIV inc or prev*	Sex specific estimates available?	Prev. of exposure	Overall prev. of HIV	Study sample size	Notes on exposure measurement
		Syphilis	RPR + TPHA								1.5%			Active infection (RPR+ & TPHA +)
**Nag VL [84] 2009**	2004	Syphilis	VDRL + TPHA	ELISA + Rapid test	UP	STI	CS	H	P	N	3.2%	22.3%	220	VDRL reactive; VDRL+ & TPHA+ reactive
**Jindal N [85] 2007**	2005	Sexual partners	Self-report	ELISA + Rapid test	PJ	STI	CS	H	P	Y	93.2%		280	Hx of multiple sex partners (lifetime)
**NFHS-3 [64] 2006**	2006	Circ./Muslim	Self-report	2 ELISAs	INDIA	General	CS	L	P	Y	12.9%	0.3%	99,838	Religion
		GUD	Self-report								8.7%			Genital ulcer symptoms in past 12 months
		Sexual partners	Self-report								9.4%			Lifetime sexual partners (1, 2-4, >5)
		Paid for sex	Self-report					L			1.1%			
**Panda S [86] 2005**	2003	Paid for sex	Self-report	2 ELISAs	TN	IDU	CS	H	P	Y	60.6%	30.1%	226	Ever had sex with FSW
**Ramesh BM [87] 2008**	2003	Sexual partners	Self-report	2 ELISAs	S.INDIA	FSW	CS	H	P	Y	33.1%	14.6%	10,096	Client volume per week (<10, >=10)
**Reynolds SJ [10] 2003**	1993-2000	HSV-2	IgG ELISA	ELISA + Rapid test	MH	STI	CHRT	H	I	N	43.0%	8.2%	2,732	Incident if enrolled sero- & sero+ at follow up
		Paid for sex	Self-report											CSW partners in past 3 mo.; FSW risk group
**Reynolds SJ [7] 2006**	1993-2000	Syphilis	RPR + TPHA; dark field microscopy if GUD	ELISA + Rapid test	MH	STI	CHRT	H	I	N	14.8%	8.2%	2,729	Incident if TPHA seroconversion, or GUD + (dark field microscopy/RPR seroconversion)
**Rodrigues JJ [88] 1995**	1993-1994	GUD	physical exam + Self-report	ELISA, Rapid, + WB	MH	STI	CS	H	P	N	52.7%	23.5%	2,790	Current genital ulcer; Hx of genital ulcer
**Samuel NM [89] 2007**	2003-2004	Circ./Muslim	Self-report	ELISA, Rapid, + WB	TN	ANC	CS	L	P	Y	3.8%	2.0%	3,715	Religion
**Sarkar K [90] 2006**	2004	Sexual partners	Self-report	ELISA + Rapid test	WB	FSW	CS	H	P	Y	18.8%	5.9%	2,076	>3 clients per day
		syphilis	TPHA + VDRL								13.6%			Recent infection if TPHA & VDRL +
**Schneider JA [91] 2010**	2004-2005	HSV-2	2 ELISAs (IgG)	ELISA , WB, p24 + pooled nucleic acid	AP	General	CS	L	P	Y	7.5%	1.9%	12,617	Lifetime infection
		Syphilis	2 ELISAs (IgG)								1.7%			Lifetime infection
**Shahmanesh M [92] 2009**	2004	GUD	Self-report	2 ELISAs	GOA	FSW	CS	H	P	Y	7.4%	23.7%	325	GUD in last 3 months
		HSV-2	IgG ELISA								56.6%			Lifetime infection
		Sexual partners	Self-report								62.2%			Number of clients in past week (0, 1-7, 7+); number of intimate non-paying partners (0, 1, >1)
		Syphilis									25.8%			
**Shepherd ME [93] 2003**	1993-2000	Sexual partners	Self-report	ELISA + Rapid	MH	STI	CHRT	H	I	Y	14.0%	7.3%	2,255	Number of 'recent' partners (0, 1, >2)
		Paid for sex	Self-report								23.0%			Recent CSW partner
**Shethwala N [94] 2009**	2005-2006	Syphilis	RPR + TPHA	ELISA + Rapid	GJ	FSW	CS	H	P	Y	11.7%	11.7%	300	RPR+ & TPHA+
**Solomon S [96] 2010**	2008	HSV-2	IgG ELISA	2 ELISAs	TN	MSM	CS	H	P	Y	25.8%	9.0%	721	lifetime infection
		Syphilis	RPR + TPPA								8.0%			RPR & TPPA +
**Solomon S [95] 1998**	1994-1995	GUD	Self-report	ELISA + WB	TN	General	CS	L	P	N	34.7%	7.3%	2,063	Hx of GUD
		Sexual partners	Self-report								48.3%			single partner vs multipartner
		Syphilis	VDRL								5.1%			VDRL test
**Talukdar A [16] 2007**	2006	Circ./Muslim	Self-report	3 Rapid ELISAs	WB	Homeless	CS	H	P	Y	22.0%	3.5%	485	Religion
		Sexual partners	Self-report								90.3%			multiple sexual partners (time period not specified)
		Paid for sex	Self-report								73.4%			visited CSW in past 6 months
		Syphilis	TRUST + TPHA								7.1%			TRUST (toludine red unheated serum test) & TPHA+

Footnote:
Circ./Muslim=male circumcision or Muslim religion; HSV-2=Herpes Simplex Virus 2; State: KN=Karnataka, MH=Maharashtra, AP=Andhra Pradesh, S.India= South India; Population type: STI=Sexually transmitted infection clinic attendees; General=general population survey, FSW=female sex worker, MSM=Men who have sex with men, ANC=antenatal clinic attendees, IDU=injecting drug users; Design: CS=cross-sectional, CHRT=cohort, CCTRL=Case-control; HIV risk group: H=high, L=low (see methods).
