# Supplementary material for: A Systematic Review and Meta-Analysis of Risk Factors for Sexual Transmission of HIV in India
Source: PLoS One. 2012 Aug 28;7(8):e44094. doi: 10.1371/journal.pone.0044094 (PMC3429412; doi:10.1371/journal.pone.0044094)
Supplement: Table S2 — Results of random effects meta-regression for six study characteristics. Footnotes: 1. State = Four large south Indian states versus all others. 2. F-test for categorical variables (DOC) [file pone.0044094.s004.doc]

**Supplemental Table 2:** Results of random effects meta-regression for six study characteristics.

| **Exposure** | **No. of studies** | **Characteristic ^1^** | **Coeff.** | ***p ^2^*** |
| --- | --- | --- | --- | --- |
|  |  |  |  |  |
| **Circumcision** | 13 | Gender (male v. female) | -0.052 | 0.852 |
| **or muslim religion** |  | HIV risk group (low v. high) | -0.259 | 0.377 |
|  |  | Study year | -0.036 | 0.329 |
|  |  | Study design (cross-sect. v. longit) | -0.104 | 0.807 |
|  |  | State | 0.351 | 0.182 |
|  |  | HIV test method | 0.209 | 0.470 |
|  |  |  |  |  |
| **Paid sex (males)** | 9 | HIV risk group (low v. high) | 0.385 | 0.528 |
|  |  | Study year | 0.011 | 0.895 |
|  |  | Study design (cross-sect. v. longit) | -0.240 | 0.728 |
|  |  | State | -0.352 | 0.628 |
|  |  | HIV test method | -0.065 | 0.915 |
|  |  |  |  |  |
| **Genital ulcer** | 14 | Gender (male v. female) | 0.616 | 0.826 |
|  |  | HIV risk group (low v. high) | -0.256 | 0.617 |
|  |  | Study year | 0.000 | 0.995 |
|  |  | Study design (cross-sect. v. longit) | -0.091 | 0.817 |
|  |  | State | 0.726 | 0.177 |
|  |  | HIV test method | 0.163 | 0.726 |
|  |  |  |  |  |
| **HSV-2** | 11 | Gender (male v. female) | -0.337 | 0.384 |
|  |  | HIV risk group (low v. high) | *1.131* | *0.017* |
|  |  | Study year | 0.071 | 0.464 |
|  |  | Study design (cross-sect. v. longit) | 0.925 | 0.260 |
|  |  | State | 0.824 | 0.347 |
|  |  | HIV test method | 0.909 | 0.080 |
|  |  |  |  |  |
| **Syphilis** | 22 | Gender (male v. female) | -0.186 | 0.562 |
|  |  | HIV risk group (low v. high) | *1.672* | *<0.0001* |
|  |  | Study year | 0.041 | 0.480 |
|  |  | Study design (cross-sect. v. longit) | 0.636 | 0.387 |
|  |  | State | 0.426 | 0.934 |
|  |  | HIV test method | 0.276 | 0.582 |
|  |  |  |  |  |
| **Multiple partners** | 24 | Gender (male v. female) | 0.326 | 0.117 |
|  |  | HIV risk group (low v. high) | *0.657* | *0.013* |
|  |  | Study year | 0.022 | 0.547 |
|  |  | Study design (cross-sect. v. longit) | -0.390 | 0.249 |
|  |  | State | 0.242 | 0.443 |
|  |  | HIV test method | *1.008* | *0.003* |

*Footnotes:*

*1. State= Four large south Indian states versus all others.*

*2. F-test for categorical variables*
